# The Progress of Psycho-Analysis
*The International Psycho-Analytical Library. No. 2: *Psycho-Analysis and the War Neuroses.* (International Psycho-Analytical Press and Geo. Allen & Unwin, Ltd. 1921.)*The International Journal of Psycho-Analysis,* Vol. I., Part 4. (International Psycho-Analytical Press, London, Vienna, New York.)


**Published:** 1921-07-16

**Authors:** 


### THE PROGRESS OF PSYCHO-ANALYSIS.*

The reception in this country of Freud's work was characterised, as readers know, by initial extreme hostility and vituperation, tempered by one or two good judges who publicly gave their adherence and then prudently held their tongues. This attitude—the general one, we mean—was, of course, fostered by contemporary bellicose sentiment, which again was fostered by the "transient era of organised insincerity" (to use an ex-Prime Minister's rather optimistic words) which then reigned over our minds, even those of the best of us. There has been a change since, a change which no doubt Freudians would term an awakening. Within three years of an eminent British alienist's pronouncement that psycho-analysis was dead in England several books on the subject appeared, and, what is more, sold in a slumping book market. Then came an international journal—its name is appended —and now just lately this symposium about the bearing of psycho-analysis on war neurosis. That had been urged as a weak point of the science, a familiar doctrine of which is the sexual origin of neurosis, whereas in the form under notice the terrors of war seemed to be the *causa efficiens*. The Freudian school's way of meeting the objection is that, owing to easily imaginable practical difficulties, very few psycho-analyses of war neurotics were made, and none published. Such examination might have revealed, as in the case of traumatic neurosis in peace time, etiological factors of a sexual nature. Moreover, they say, it transpires with great regularity that these victims of war were originally "labile" persons, especially so as regards their sexuality. In any case, war experience has led academic neurologists in most countries to regard the causation of traumatic neurosis in a psychological light. The rival orthodox theory is that these disturbances are to be explained as a failure of maintenance of equilibrium (ill-sounding word in a theory!) between instinctive tendencies and the forces by which they are controlled. Hysterical paralysis is thus a reversion to the primitive instinct of immobility in the face of unescapable danger, as seen in the phenomenon of "shamming dead." Even trembling may be explained thus, from the unopposed action of antagonisers of fear-paralysed muscles. Such primitive instincts, now no longer biologically useful, are brought again to the surface in the neurosis. But Freud had already drawn attention to the repressive character of neurotic symptoms, and, indeed, his whole theory of the unconscious, with its primitive emotional traits, bears strongly on atavism. It is well known, too, that the connotation Freud attaches to the term sexuality is a very wide one, taking in affection, and, indeed, mere egotism or self-love. In an opening contribution Freud himself opines that the especial sexual factor in war neurosis will prove to be this very self-love or narcissism. As is often the case in a bundle of essays from different hands, some contradictions occur. One writer, says that, although hypnosis may remove a symptom, another will take its place; another describes hypnotic procedures as leading to a true *catharsis*. Simmel, of Berlin, seems to have carried out a fair amount of psycho-analytic therapy, which indeed the Medical Department of the Prussian State Ministry was organising when political changes interrupted. Abreaction was aided by the use of clothed dummy figures to reproduce war experience. Dr. Ernest Jones, on the other hand, thinks it superfluous in the large majority of cases. The truth will no doubt settle these slight differences, as it is already deciding the much greater strife we noticed to begin with. It is a happy thought that truth is immortal.

The journal spoken of seems a well found publication on the model of the previous Continental ones. An article which will interest lay as well as medical readers (a feature, this, of psycho-analysis generally) deals with a clear anticipation of some of Freud's doctrines by no other than the critic Hazlitt, the friend of Coleridge. As briefly noticed already in Mordell's "The Erotic Motive in Literature," Hazlitt remarks of dreams that "our tacit and almost unconscious sentiments" are discovered in dreams. "When awake we check these rising thoughts, and fancy we have them not. In dreams, when we are off our guard, they return securely and unbidden." In this passage and others there is clear premonition of what Freudians now familiarly call the *unc*. The fact is not surprising. In this journal, indeed, the point has been made that the generalising power of the great critic is a scientific, not an artistic, trait, very akin to the faculty of the investigator, the "superb guessing" of a Newton. This "Note on Hazlitt" would rejoice any editor's heart, but one would have thought its predecessor should have turned his stomach. The Viennese journal *Imago* is, so to say, the literary supplement to Freudian publications, dealing with the bearing of the unconscious on the productions of imaginative authors. Here that most difficult and risky business, traffic between the literary and scientific camps, is carried on, often (as, for instance, by Rank) with brilliant success. Always the level is far above that of this American contribution. To quote famous poems *in extenso*, adding three or four lines of bald comment, and to write on the subject of literature in prose radically bad, is to the sensitive nothing short of an offence. Young papers, like new brooms, should be efficient, and the academic critic who always has his knife half-drawn against psycho-analysis here finds the guard open. His thrusts will, however, do no permanent damage. To have a desperately sickly infancy, a puny childhood, a magnificent adolescence, and an immortal old age is the very mark, the very way, of what is both new and true.

* The International Psycho-Analytical Library. No. 2: *Psycho-Analysis and the War Neuroses.* (International Psycho-Analytical Press and Geo. Allen & Unwin, Ltd. 1921.)

*The International Journal of Psycho-Analysis*, Vol. I., Part 4. (International Psycho-Analytical Press, London, Vienna, New York.)

